# Elimination prospects of the Dutch HIV epidemic among men who have sex with men in the era of preexposure prophylaxis

**DOI:** 10.1097/QAD.0000000000002050

**Published:** 2018-11-07

**Authors:** Ganna Rozhnova, Janneke Heijne, Daniela Bezemer, Ard van Sighem, Anne Presanis, Daniela De Angelis, Mirjam Kretzschmar

**Affiliations:** a Julius Center for Health Sciences and Primary Care, University Medical Center Utrecht, Utrecht; b Centre for Infectious Disease Control, National Institute of Public Health and the Environment, Bilthoven; c Stichting HIV Monitoring, Amsterdam, The Netherlands; d Medical Research Council Biostatistics Unit, Cambridge Institute of Public Health, University of Cambridge, Cambridge, UK.

**Keywords:** HIV elimination, HIV prevalence, mathematical modeling, men who have sex with men, preexposure prophylaxis, preexposure prophylaxis coverage

## Abstract

**Objective:**

Preexposure prophylaxis (PrEP) is a promising intervention to help end the HIV epidemic among men who have sex with men (MSM) in the Netherlands. We aimed to assess the impact of PrEP on HIV prevalence in this population and to determine the levels of PrEP coverage necessary for HIV elimination.

**Design and methods:**

We developed a mathematical model of HIV transmission in a population stratified by sexual risk behavior with universal antiretroviral treatment (ART) and daily PrEP use depending on an individual's risk behavior. We computed the effective reproduction number, HIV prevalence, ART and PrEP coverage for increasing ART and PrEP uptake levels, and examined how these were affected by PrEP effectiveness and duration of PrEP use.

**Results:**

At current levels of ART coverage of 80%, PrEP effectiveness of 86% and PrEP duration of 5 years, HIV elimination required 82% PrEP coverage in the highest risk group (12 000 MSM with more than 18 partners per year). If ART coverage increased by 9%, the elimination threshold was at 70% PrEP coverage. For shorter PrEP duration and lower effectiveness elimination prospects were less favorable. For the same number of PrEP users distributed among two groups with highest risk behavior, prevalence dropped from the current 8 to 4.6%.

**Conclusion:**

PrEP for HIV prevention among MSM could, in principle, eliminate HIV from this population in the Netherlands. The highest impact of PrEP on prevalence was predicted when ART and PrEP coverage increased simultaneously and PrEP was used by the highest risk individuals.

## Introduction

As stated by the Joint United Nations Programme on HIV/AIDS [1], their aim in the post-2015 era is to end the AIDS epidemic by 2030. To achieve elimination, the ‘90–90–90’ targets for HIV treatment scale-up were announced [1]. By 2020, 90% of all people living with HIV must be diagnosed, 90% of people with a HIV diagnosis must receive antiretroviral treatment (ART), and 90% of treated people must be virally suppressed. Three years before 2020, the Netherlands has exceeded these targets for men who have sex with men (MSM) with the most recent estimates of 90–94–96 percentage in the corresponding groups [2]. However, we are still far from stopping HIV transmission, especially in MSM who accounted for 68% of new HIV diagnoses in 2016 [2].

At present preexposure prophylaxis (PrEP) is considered to be one of the most promising interventions to help end the HIV epidemic in this population [3,4]. Daily or intermittent PrEP uptake was shown to be effective in preventing HIV transmission among MSM in trials [5,6], cohort [7] and clinical practice studies [8,9]. The body of modeling work assessing PrEP impact in different settings has been growing in the last several years. Research has mostly focused on the cost-effectiveness of PrEP in the context of the Netherlands [10] and other countries [11–13]. Modeling studies predicted the pivotal role of PrEP in reducing HIV incidence in MSM, particularly in the United Kingdom [14]. However, these studies mainly investigated short-term impact of PrEP on HIV transmission by examining reduction in HIV incidence. The question of the possibility of HIV elimination by PrEP and the levels of PrEP uptake necessary to achieve it has not been addressed systematically in the literature.

The current guideline by the Dutch Association of HIV-treating Physicians recommends PrEP use by MSM at high risk of HIV acquisition [15]. The high price of this preventive medication has been the main limitation for a large-scale implementation of PrEP intervention in the Netherlands. PrEP coverage among MSM is below 10% [16]. However, changes in PrEP uptake are expected to occur as the price of PrEP has recently dropped by 80% [17]. The knowledge to aid PrEP rollout in the Netherlands will be provided by a PrEP study known as the Amsterdam PrEP Project [18] running from June 2015. As part of Amsterdam PrEP Project, real-world data on the uptake by high-risk MSM of daily and intermittent PrEP, medication adherence and change in risk behavior when taking PrEP will be collected by the Public Health Service Amsterdam until December 2018 [18].

Our aim was to investigate the potential impact of PrEP on the HIV epidemic among MSM in the Netherlands using a mathematical transmission model. The model assumed universal ART of the population and daily PrEP use depending on an individual's risk behavior. We estimated the reduction in HIV prevalence for increasing levels of PrEP uptake and determined which levels of PrEP uptake, effectiveness and duration would be necessary for HIV elimination.

## Methods

### Model formulation and assumptions

The mathematical model described HIV transmission among MSM through unprotected sex. The model was based on our previous model [19] which we extended to include PrEP. We stratified the population into four risk groups by the average number of new sexual partners per year. The model allowed to vary mixing between the risk groups. As estimates of the degree of assortative mixing in the population of MSM in the Netherlands are not available, we assumed intermediate mixing. Intermediate mixing implies that half of partnerships are formed within the same risk group and the remaining half are formed with individuals from other risk groups, proportionally to the number of partnerships offered by those risk groups. Average duration of sexual activity was 45 years.

The compartments in the model represented individuals who were susceptible, infected in four disease stages (primary infection, chronic infection, AIDS stage with onset of severe symptoms, terminal AIDS stage without sexual activity) and those treated in four stages. Infection stages had different infectivities and duration. Infectivity of individuals on ART was reduced and did not differ between the stages. The model assumed universal ART and that treatment uptake was the same in all infection stages and risk groups. A small percentage of treated individuals could drop off ART and return to the respective infected stage.

We considered two scenarios for daily PrEP use. In the first scenario, PrEP could be taken by the highest risk individuals only. In real world, PrEP will probably be more commonly used in the highest risk population but likely in other risk groups as well. For this reason in our second scenario, PrEP was taken by individuals in two groups with highest risk. When PrEP was taken by the two highest risk groups we kept PrEP uptake rate equal in both groups. To be able to make a comparison of the two scenarios we adjusted the rate of PrEP uptake in the second scenario so that the total number of individuals on PrEP in the two highest risk groups was the same as the number of PrEP users in the highest risk group in the first scenario. The average duration of PrEP use and PrEP effectiveness could be varied. The effectiveness was defined as the relative reduction in the rate of HIV infection due to PrEP use [5]. At 100% effectiveness PrEP users never got infected. At 0% effectiveness PrEP users got infected with the same rate as non-PrEP users. Infectivity of individuals who acquired HIV while taking PrEP was assumed to be lower than infectivity of MSM who did not take PrEP. As individuals taking PrEP are regularly tested for HIV (every 3 months in the AMPrEP study [18]), in the model infected PrEP users started treatment during primary infection.

The model was implemented as a system of ordinary differential equations in the Mathematica software by Wolfram Research (version 10.0.2). The detailed model description and equations are given in the Supplementary Material.

### Effective reproduction number

We computed the effective reproduction number, *R*_*e*_, using next generation matrix theory described in detail elsewhere [20,21]. This quantity served as a measure of HIV transmission potential in the presence of ART and PrEP and was used to define the conditions for HIV elimination. Mathematically, *R*_*e*_ is defined as the average number of secondary infections caused by a typical infectious individual in a population, where intervention measures are implemented. Although *R*_*e*_ is a mathematical concept used in modeling studies, it is useful for HIV prevention and elimination policy planning. Specifically, *R*_*e*_ allows to quantify the effort necessary for eliminating a disease. In theory, disease elimination occurs if *R*_*e*_ is below 1. In what follows, we referred to parameters for which HIV elimination occurred as parameters for which *R*_*e*_ = 1, that is at the threshold of elimination.

### Model outputs

We numerically solved the model equations to determine three steady-state quantities: HIV prevalence, PrEP coverage per risk group and population level ART coverage. HIV prevalence was computed as the percentage of HIV infected in the population in the steady state. PrEP coverage was defined per risk group as the percentage of HIV-negative individuals who were on PrEP in that group in the steady state. Population level ART coverage was defined as the percentage of HIV-infected individuals who were on treatment in the steady state.

### Model parameters

Most parameter values were taken from the existing literature (Supplementary Table 1). Behavioral parameters were estimated from data. The average number of new partners per year was determined from sexual behavior data on the self-reported number of sexual partners in the last 6 months for MSM in the Netherlands by Rutgers World Population [22], using the method described in detail in Rozhnova *et al.*[19]. In this method, we used the actual number of partners for respondents who reported inconsistent condom use. The respondents who always used condoms were assumed to have zero sexual partners. We used population stratification by risk from Rozhnova *et al.*[19] in which three groups with highest risk were grouped into one. The resulting percentages of MSM in the four risk groups were 45.1, 35.3, 12.5 and 7.1%. The average numbers of new partners per year (range) were 0.13 (0–0.45), 1.43 (0.45–3.35), 5.44 (3.35–8.88) and 18.21 (8.88–500), respectively.

Default PrEP parameters were effectiveness of 86% [5,6] and duration of 5 years [10]. We assumed that infectivity of individuals on PrEP was half the infectivity of non-PrEP users and that 95% of individuals who got infected while taking PrEP started ART within 1 year. These assumptions were relaxed in the sensitivity analyses of the model (refer to Fig. 2 in the Supplementary Material).

We presented our results in terms of the model parameters such as annual ART and PrEP uptake percentages (range 0–100%). The former and the latter were defined respectively as the percentage of infected individuals starting ART and the percentage of HIV-negative individuals initiating PrEP within 1 year. For given annual PrEP and ART uptake percentage we computed steady-state ART and PrEP coverage as the model output.

Annual ART uptake percentage and probability of transmission per partnership were free parameters in the model. We fixed annual ART uptake at 30% and probability of transmission per partnership at 0.25 in the baseline model scenario without PrEP to describe the current state of the Dutch HIV epidemic among MSM. HIV prevalence and ART coverage in the model (before PrEP) were about 8% (estimate for MSM in the Netherlands [23]) and 80% (estimate by HIV Monitoring Foundation [2]), respectively. Population size in the absence of interventions was 210 000 individuals.

## Results

**Fig. 1:**
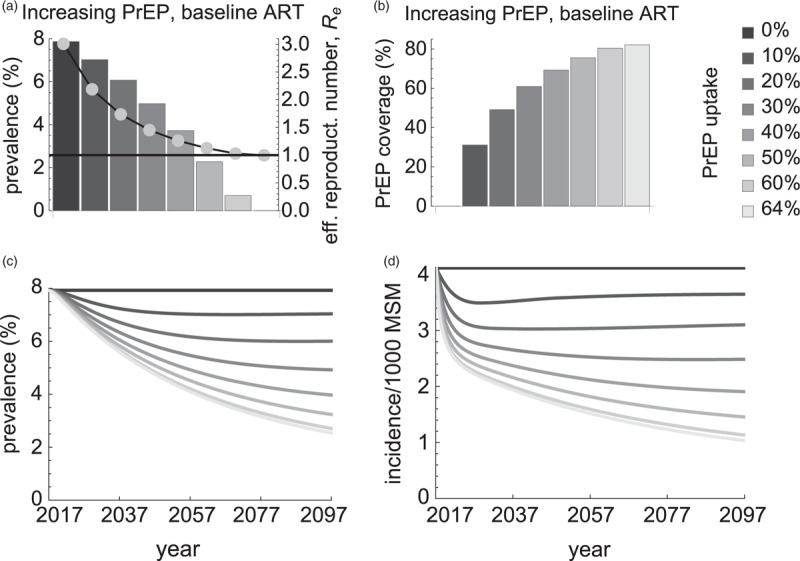
Impact of preexposure prophylaxis uptake in the highest risk group.

PrEP use by highest risk individuals was an efficient way to reduce steady-state HIV prevalence and to drive HIV to elimination (Fig. 1). Assuming baseline ART uptake of 30%, steady-state prevalence dropped from 8 to 6.1% if PrEP uptake was 20% annually, and from 8 to 3.7% for 40% uptake (Fig. 1a). The elimination (zero prevalence and *R*_*e*_ just below 1 in Fig. 1a) was reached at PrEP uptake of 64% which translated into 82% PrEP coverage (Fig. 1b). As in the steady state the highest risk group was about 7% of the population, this coverage implied that 5.7% of all MSM had to be taking PrEP to achieve elimination. For a population of 210 000 MSM about 12 000 individuals with more than 18 sexual partners per year had to use PrEP to eliminate HIV.

(a) Steady-state HIV prevalence (bars, the left *y*-axis) and effective reproduction number *R*_*e*_ (dots, the right *y*-axis), (b) preexposure prophylaxis coverage in the highest risk group, (c and d) time-dependent dynamics of HIV prevalence and incidence for increasing levels of preexposure prophylaxis uptake and baseline antiretroviral treatment. Preexposure prophylaxis uptake (bar color) denoted the percentage of highest-risk MSM initiating preexposure prophylaxis within 1 year. Preexposure prophylaxis coverage was computed as the steady-state percentage of HIV-negative MSM who were on preexposure prophylaxis in the highest risk group. The black line in part (a) is *R*_*e*_ = 1. Below this value of *R*_*e*_ HIV prevalence became zero indicating HIV elimination. The elimination occurred when preexposure prophylaxis uptake exceeded 64%.

The analysis in Fig. 1a referred to values of the steady-state prevalence. In practice, reaching elimination, viz the steady state with zero HIV prevalence and incidence, required a long time (Fig. 1c and d). At the elimination threshold (PrEP uptake of 64%), HIV prevalence and incidence dropped by half 40 and 15 years after initiation of the PrEP intervention, respectively. After 80 years, HIV prevalence was still 2.5% and HIV incidence was reduced by 75%. Given a time frame, the higher PrEP uptake was the larger impact it had on HIV prevalence. Higher PrEP uptakes would be necessary given a shorter time frame to achieve a certain reduction in HIV prevalence (refer to Supplementary Fig. 3).

**Fig. 2:**
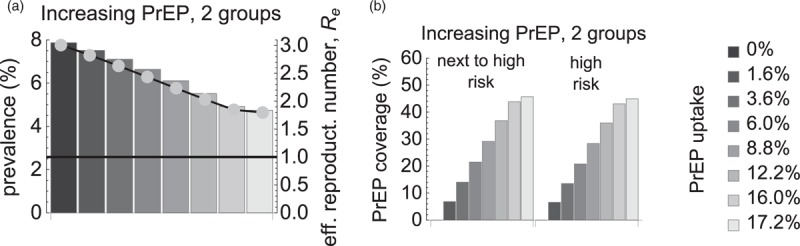
Impact of preexposure prophylaxis uptake in two groups with highest risk.

We compared the above scenario with the scenario in which PrEP was targeted to individuals in the two highest risk groups (Fig. 2). The total number of individuals on PrEP was the same in both scenarios, ranging from about 1200 to 12 000 MSM for the range of PrEP uptakes used in Figs. 1 and 2. As expected, in the second scenario the same number of PrEP users was achieved at lower PrEP uptake because PrEP was distributed among individuals in the two highest risk groups instead of the highest risk individuals only (compare Figs. 1 and 2 by bar color). In this scenario, steady-state HIV prevalence could be reduced by PrEP from 8 to 4.6% and *R*_*e*_ from 3 to 1.8 (Fig. 2a). As compared with the first scenario in which our model predicted HIV elimination (Fig. 1a), in the second scenario the elimination by PrEP was not feasible in the full range of explored parameters.

(a) Steady-state HIV prevalence (bars, the left *y*-axis) and effective reproduction number *R*_*e*_ (dots, the right *y*-axis), (b) preexposure prophylaxis coverage in the next to highest and in the highest risk groups for increasing levels of preexposure prophylaxis uptake and baseline antiretroviral treatment. Preexposure prophylaxis uptake (bar color) was equal in both groups and the total number of preexposure prophylaxis users in the two groups was the same as the number of preexposure prophylaxis users in the highest risk group in Fig. 1. Figures 1 and 2 can be compared by bar color. Preexposure prophylaxis coverage was computed per risk group as the steady-state percentage of HIV-negative MSM who were on preexposure prophylaxis in that group. The values of *R*_*e*_ were always far above 1 (black line in part b) indicating that elimination by preexposure prophylaxis was not feasible.

**Fig. 3:**
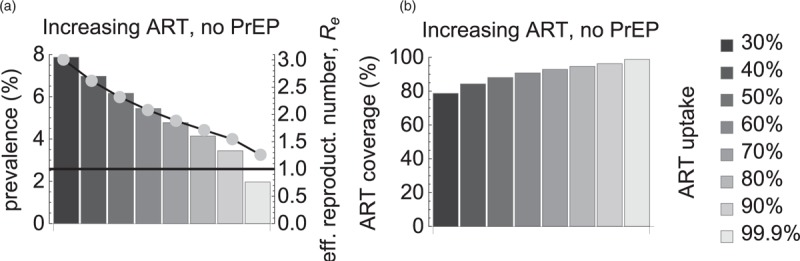
Impact of antiretroviral treatment uptake if preexposure prophylaxis is unavailable.

Significantly, we came to the same conclusion of impossibility of elimination in a scenario with increasing ART uptake levels and no PrEP (Fig. 3). If ART uptake increased from baseline 30 to 99.9%, prevalence decreased from 8 to 2% and *R*_*e*_ decreased from 3 to 1.3 (Fig. 3a). For this range of uptakes the model predicted an increase in ART coverage from baseline 80 till 99% (Fig. 3b) but still no elimination.

(a) Steady-state HIV prevalence (bars, the left *y*-axis) and effective reproduction number *R*_*e*_ (dots, the right *y*-axis), (b) antiretroviral treatment coverage in the population for increasing levels of antiretroviral treatment uptake. Antiretroviral treatment uptake (bar color) denoted the percentage of MSM initiating antiretroviral treatment within 1 year. Antiretroviral treatment coverage was computed as the steady-state percentage of HIV-infected population on antiretroviral treatment. The black line in part (a) is *R*_*e*_ = 1. As *R*_*e*_ never decreased below 1, eliminating HIV by antiretroviral treatment was not feasible in this scenario.

**Fig. 4:**
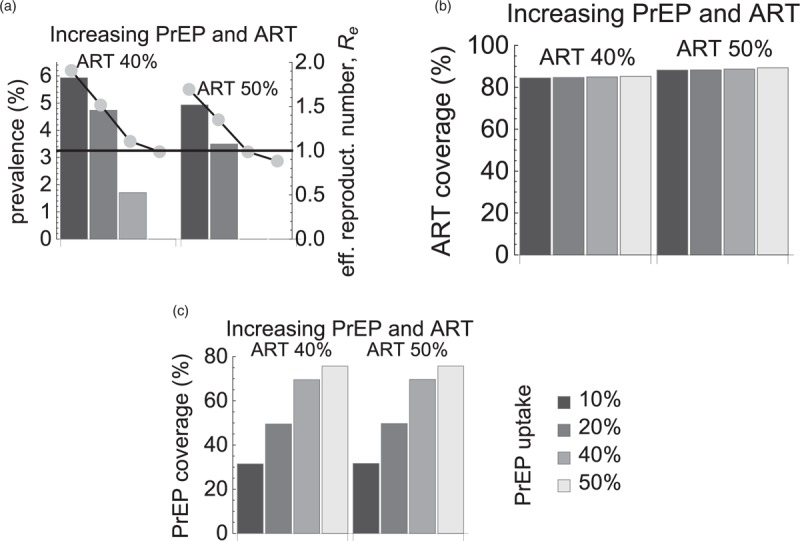
Impact of increasing antiretroviral treatment levels in the entire population and preexposure prophylaxis uptake in the highest risk group.

As ART coverage has been increasing in the Netherlands, we further extended our analysis of the first scenario with PrEP uptake in the highest risk group assuming that ART uptake levels increased as well (Fig. 4). As expected, the impact of both interventions was higher than the impact of PrEP alone in the sense that higher ART uptake required lower PrEP uptake to achieve the same reduction in the steady-state HIV prevalence (compare Figs. 1 and 4 by PrEP uptake level). If ART uptake increased from baseline 30 to 40 and 50%, elimination could be achieved at PrEP uptake of 50 and 40%, respectively (Fig. 4a). In terms of intervention coverage (Fig. 4b and c), HIV was eliminated at ART coverage of 85% and PrEP coverage of 76%. If ART coverage increased to 89%, then PrEP coverage had to be 70% for elimination.

(a) Steady-state HIV prevalence (bars, the left *y*-axis) and effective reproduction number *R*_*e*_ (dots, the right *y*-axis), (b) antiretroviral treatment coverage in the population and (c) preexposure prophylaxis coverage in the highest risk group for increasing levels of preexposure prophylaxis uptake (bar color). At 40 and 50% antiretroviral treatment uptake the model predicted HIV elimination (*R*_*e*_ = 1 – black line) at 50 and 40% preexposure prophylaxis uptake (bar color), respectively. In terms of coverage, HIV was eliminated at 85% antiretroviral treatment coverage and 76% preexposure prophylaxis coverage, or at 89% antiretroviral treatment coverage and 70% preexposure prophylaxis coverage.

**Fig. 5:**
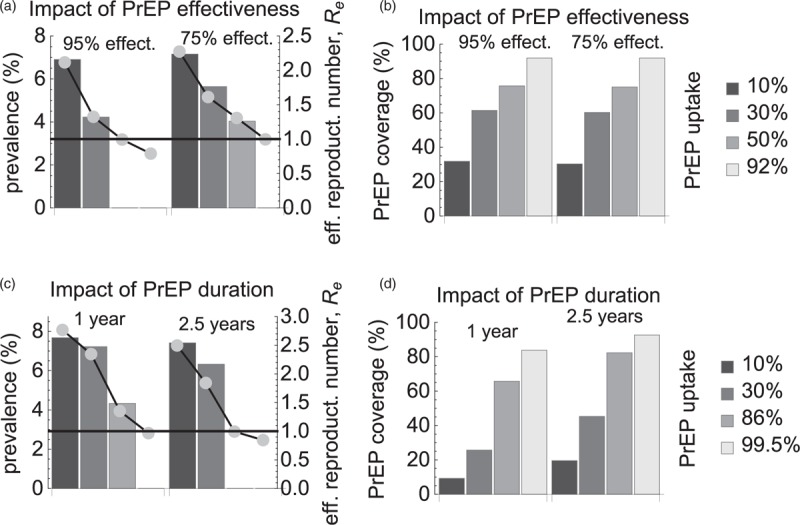
Impact of preexposure prophylaxis effectiveness and duration of taking preexposure prophylaxis in the highest risk group.

The predictions of our model were so far based on the assumption of 86% PrEP effectiveness and an average duration of taking PrEP of 5 years. In Fig. 5, we relaxed these assumptions for the first scenario, changing one of these parameters and keeping the other parameter fixed at its default value. When we increased effectiveness to 95%, *R*_*e*_ became less than 1 at 50% PrEP uptake (76% PrEP coverage). If effectiveness decreased to 75%, the elimination threshold was at 92% PrEP uptake (92% coverage). For lower uptakes, PrEP led to a significant reduction in the steady-state prevalence. Shorter use of PrEP (Fig. 5c and d) made elimination prospects even less favorable. If MSM stayed on PrEP for 2.5 years, annual PrEP uptake had to be at least 86% to get to zero steady-state prevalence. For the duration of 1 year, PrEP uptake had to exceed 99.5% for this to happen. The PrEP coverage for HIV elimination with PrEP duration of 2.5 and 1 years was 82 and 84%, respectively.

(a and c) Steady-state HIV prevalence (bars, the left *y*-axis) and effective reproduction number *R*_*e*_ (dots, the right *y*-axis), (b and d) preexposure prophylaxis coverage in the highest risk group for increasing levels of preexposure prophylaxis uptake (bar color). In parts (a and b), two sets of bars corresponded to preexposure prophylaxis effectiveness of 95 and 75%, whereas preexposure prophylaxis duration was fixed at the default value of 5 years. In parts (c and d), two sets of bars corresponded to preexposure prophylaxis duration of 1 and 2.5 years, whereas preexposure prophylaxis effectiveness was fixed at the default value of 86%.

## Discussion

The use of PrEP among MSM in the Netherlands was found to be an effective intervention for reducing HIV prevalence and eventually driving HIV to elimination. The precise amount of expected reduction depended, however, on how PrEP was distributed in the population. PrEP uptake by the highest risk individuals was the most promising intervention with HIV elimination achieved at PrEP coverage of 82% (about 5.7% of all MSM or 12 000 with more than 18 new partners per year). The time it took to reach elimination after the introduction of PrEP was rather long. For PrEP coverage at the elimination threshold, HIV prevalence and incidence decreased by half within 40 and 15 years, and it took more than 80 years for them to drop to zero.

If the number of PrEP users was the same as in the initial scenario but they were distributed among the two highest risk groups then the impact of PrEP on prevalence was much less. For the range of explored parameters, the maximum prevalence reduction (from 8 to 4.6%) for PrEP use in two groups was observed when PrEP coverage among the next to highest and highest risk individuals reached 46%. Although there were significantly more PrEP users in the next to highest risk group they had lower numbers of partners and thus contributed less to transmission. This suggests that PrEP should be primarily targeted at the highest risk individuals, and a large PrEP effect on the population level HIV prevalence cannot be achieved by an extensive PrEP use in individuals with lower risk.

Given that ART coverage among MSM in the Netherlands has been increasing for years [2,24], we extended our model predictions for the scenario with PrEP uptake by the highest risk group and increasing ART uptake levels. If ART coverage increased by 9% from the current levels, the elimination was feasible at PrEP coverage of about 70%. On the other hand, our model predicted that increasing treatment uptake alone would be insufficient to reach elimination. These results agree with those of [14,25,26] who showed that a significant reduction in HIV transmission could not be achieved without PrEP.

PrEP can be administered daily or on demand (before and after a sexual contact) [3], with both regimens demonstrating an effectiveness of 86% [5,6] that we used in our analysis. Our choice of modeling daily PrEP only was motivated by the interim findings on the preference of PrEP regimes in the AMPrEP project, which showed that 72% of participants chose to start daily PrEP [3,27]. We also obtained predictions of the model for other values of effectiveness and varying duration of using PrEP to reflect a real world setting where individuals are themselves responsible for obtaining and taking PrEP drugs. As expected, HIV elimination prospects were less favorable for lower effectiveness and shorter duration on PrEP but the reduction in steady-state prevalence was still significant.

The strength of our approach is that it can be used to investigate the feasibility of HIV elimination and to determine the levels of PrEP and ART coverage necessary to achieve it. Moreover, the model is very general and could be applied for MSM populations in other Western countries such as the United Kingdom, Switzerland or the USA.

Our model had several simplifying assumptions that are likely to affect the inferences made in this study. We calibrated ART uptake using the estimate of ART coverage in the population of MSM as a whole. For this reason, we assumed ART uptake independent of risk level. In practice, higher risk individuals could test for HIV more frequently and start ART sooner upon HIV diagnosis [28]. In case of heterogeneous ART uptake in the future we expect that HIV elimination would be easier to achieve because the effective reproduction number is lower than in the case of homogeneous ART uptake [19].

Our model did not take some details of partnership dynamics into account that could influence the impact of PrEP on HIV prevalence. We assumed that taking PrEP will not affect the contribution of concurrent partnerships in the population, so concurrency effects are present before and after implementation of intervention strategies. In a situation with serosorting, PrEP use would be more effective, if only used in serodiscordant partnerships. We also expect that if mixing would be changed from intermediate to proportionate (assortative), HIV would be more (less) evenly distributed among risk groups [19], and providing HIV to high-risk individuals would be less (more) effective.

The model also assumed that risk behavior of individuals did not change with time. Changes in risk behavior can have a very important effect on HIV transmission. For example, short-term increases in risk behavior known as episodic risk were shown to greatly affect the prospects of HIV elimination by test-and-treat [29]. The effect of changing risk groups may be the replenishment of the high-risk group with susceptible individuals, which leads to an increase in transmission, and the diffusion of HIV infection into low-risk groups by HIV-infected men moving to lower risk levels. In reality, use of PrEP should be targeted as much as possible to persons and time periods, in which increased risk of HIV acquisition occurs, for example at persons with high rate of partner change and low condom use. PrEP will be more effective if it is taken before the onset of such a period, so in that sense our analysis can be viewed as providing a minimal estimate for impact of PrEP on HIV prevalence.

Finally, we did not consider a possible increase in risk behavior induced by PrEP initiation. These behavioral changes known as risk compensation were observed in Dutch MSM after ART became widely available in 1996 [30–33] and could occur upon PrEP implementation as well. A recent study based on a stochastic network-based mathematical model of HIV transmission among MSM in the United States concluded that risk compensation is unlikely to decrease the preventive impact of PrEP [34].

In conclusion, our analysis suggests that PrEP for HIV prevention among MSM could, in principle, eliminate HIV from this population in the Netherlands. To achieve elimination, public health services should target PrEP to individuals with highest risk behavior. The current level of PrEP uptake by the Dutch MSM may reduce HIV transmission but is insufficient to make a significant impact on the epidemic.

## Acknowledgements

The authors gratefully acknowledge funding by the Aidsfonds Netherlands, grant number 2013030. The authors are thankful to Prof Roel Coutinho for valuable comments on the article. A.P. and D.D.A. were supported by the Medical Research Council (Unit programme number MC_UU_00002/11).

### Conflicts of interest

There are no conflicts of interest.

## Supplementary Material

Supplemental Digital Content
