# Supplementary material for: Elimination prospects of the Dutch HIV epidemic among men who have sex with men in the era of preexposure prophylaxis
Source: AIDS. 2018 Nov 7;32(17):2615–23. doi: 10.1097/QAD.0000000000002050 (PMC6221378; doi:10.1097/QAD.0000000000002050)
Supplement: Supplemental Digital Content [file aids-32-2615-s001.pdf]

Supplementary Material

Elimination prospects of the Dutch HIV epidemic among men who have sex with men in the era of pre-exposure prophylaxis

Ganna Rozhnova[1], *Janneke Heijne, Daniela Bezemer, Ard van Sighem, Anne Presanis, Daniela De Angelis, and Mirjam E. Kretzschmar*

1 Model formulation

The mathematical model was based on our previous model [1] which we extended to include pre-exposure prophylaxis (PrEP) (see Figure 1). HIV stages were denoted using index k: $k = 1$ (primary infection), $k = 2$ (chronic infection), $k = 3$ (AIDS stage with onset of severe symptoms), $k = 4$ (AIDS stage where individuals are not sexually active). The population of size $N(t)$ was stratified into four risk groups with sizes $N_l(t)$ by the average number of new sexual partners per year c_l. We denoted risk groups using index $l = 1, 2, 3, 4$ in the order of increasing risk, i.e. group $l = 1$ was comprised of lowest risk individuals and group $l = 4$ consisted of highest risk individuals. Individuals did not change their risk behavior. The model was formulated as a system of differential equations for the number of individuals in different classes as follows

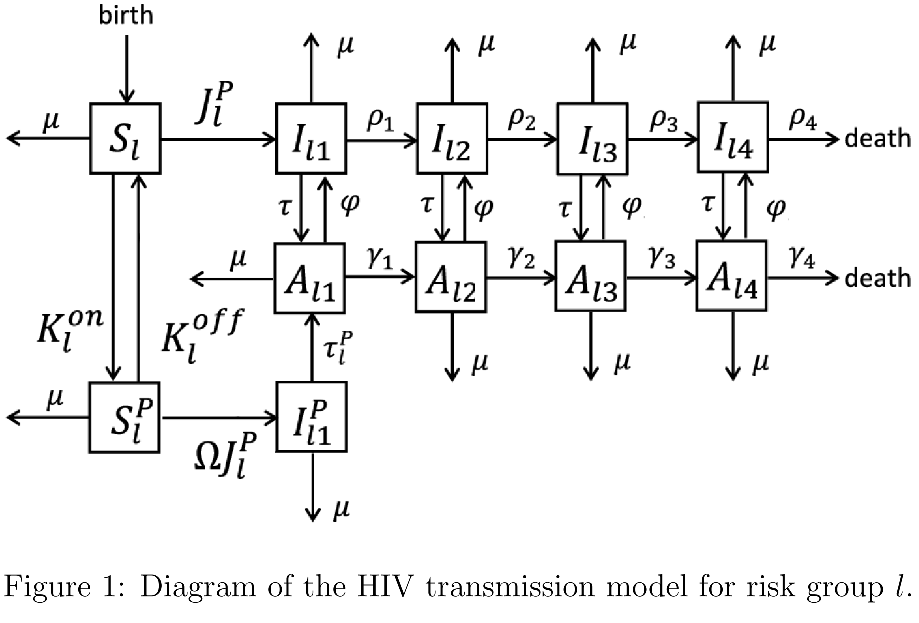

Figure 1: Diagram of the HIV transmission model for risk group l.

[1]corresponding author: G.Rozhnova@umcutrecht.nl

$$
\begin{aligned}
\frac{\mathrm{d}S_l(t)}{\mathrm{d}t} &= \mu N_0 q_l - \mu S_l(t) - J_l^P(t)S_l(t) - K_l^{on}S_l(t) + K_l^{off}S_l^P(t), \qquad (1)\\
\frac{\mathrm{d}S_l^P(t)}{\mathrm{d}t} &= -\mu S_l^P(t) - \Omega J_l^P(t)S_l^P(t) + K_l^{on}S_l(t) - K_l^{off}S_l^P(t),\\
\frac{\mathrm{d}I_{l1}(t)}{\mathrm{d}t} &= J_l^P(t)S_l(t) - (\mu+\rho_1+\tau)I_{l1}(t) + \phi A_{l1}(t),\\
\frac{\mathrm{d}I_{l1}^P(t)}{\mathrm{d}t} &= \Omega J_l^P(t)S_l^P(t) - \mu I_{l1}^P(t) - \tau_l^P I_{l1}^P(t),\\
\frac{\mathrm{d}I_{lk}(t)}{\mathrm{d}t} &= \rho_{k-1}I_{l,k-1}(t) - (\mu+\rho_k+\tau)I_{lk}(t) + \phi A_{lk}(t),\\
\frac{\mathrm{d}A_{l1}(t)}{\mathrm{d}t} &= \tau I_{l1}(t) - (\mu+\gamma_1+\phi)A_{l1}(t) + \tau_l^P I_{l1}^P(t),\\
\frac{\mathrm{d}A_{lk}(t)}{\mathrm{d}t} &= \tau I_{lk}(t) + \gamma_{k-1}A_{l,k-1}(t) - (\mu+\gamma_k+\phi)A_{lk}(t),
\end{aligned}
$$

where $k = 2, 3, 4$ and $l = 1, 2, 3, 4$. $S_l(t)$ and $S_l^P(t)$ denote the number of individuals who are susceptible and who are susceptible and take PrEP. $I_{lk}(t)$ is the number of individuals who are infected. $I_{l1}^P(t)$ is the number of individuals infected while taking PrEP. $A_{lk}(t)$ is the number of individuals on antiretroviral treatment (ART).

The force of infection (per year) in group l was given by

$$
J_l^P(t) = \lambda c_l \sum_{l'=1}^{4} M_{ll'}(t)\left[\epsilon^P \frac{I_{l'1}^P(t)}{N_{l'}(t)} + \sum_{k=1}^{4}\left(h_k \frac{I_{l'k}(t)}{N_{l'}(t)} + \epsilon \frac{A_{l'k}(t)}{N_{l'}(t)}\right)\right], \qquad (2)
$$

where $N_l(t) = S_l(t) + S_l^P(t) + \sum_{k=1}^{4}[I_{lk}(t) + A_{lk}(t)] + I_{l1}^P(t)$ is the population size in group l. The total population size can be expressed as $N(t) = \sum_{l=1}^{4} N_l(t)$. The force of infection takes into account that infectivities of untreated individuals, h_k, depend on infection stage $k = 1, 2, 3, 4$. Infectivity of individuals on ART, ϵ, is stage independent. We further assumed that individuals on PrEP who get infected may have lower viral loads levels and thus lower infectivity in the primary stage, ϵ^P, than individuals who don't take prophylaxis, h_1, $\epsilon^P \leq h_1$. The force of infection depends also on the transmission probability per partnership, λ, the average number of new partners per year, c_l ($l = 1, 2, 3, 4$), and mixing between susceptible MSM in group l and infected MSM in group $l' = 1, 2, 3, 4$. Mixing is described by a matrix with the elements $M_{ll'}(t)$

$$
M_{ll'}(t) = \omega \frac{c_{l'}N_{l'}(t)}{\sum_{l''=1}^{4} c_{l''}N_{l''}(t)} + (1-\omega)\delta_{ll'}, \qquad (3)
$$

where $\delta_{ll'} = 1$ if $l = l'$ and $\delta_{ll'} = 0$ otherwise. Mixing parameter $\omega \in [0, 1]$ allows to change mixing from assortative ($\omega = 0$) to proportionate ($\omega = 1$). Values of ω between 0 and 1 correspond to intermediate levels of mixing.

2 Model outputs

The outputs of the model were HIV prevalence, PrEP coverage per risk group and population level ART coverage, all computed at the steady state of Eqs. 1. The steady states were obtained numerically in Mathematica (version 10.0.2).

HIV prevalence was computed as $\left[\sum_{l=1}^{4}\sum_{k=1}^{4}\left(I_{lk}^{*}+A_{lk}^{*}\right)+\sum_{l=1}^{4}I_{l1}^{P*}\right]/\sum_{l=1}^{4}N_{l}^{*}$, where * refers to the steady state values of the variables. Population level ART coverage was computed as $\sum_{l=1}^{4}\sum_{k=1}^{4}A_{lk}^{*}/\left[\sum_{l=1}^{4}\sum_{k=1}^{4}\left(I_{lk}^{*}+A_{lk}^{*}\right)+\sum_{l=1}^{4}I_{l1}^{P*}\right]$. PrEP coverage in risk group l was computed as $\left(S_{l}^{P*}+I_{l1}^{P*}\right)/\left(S_{l}^{P*}+I_{l1}^{P*}+S_{l}^{*}\right)$.

3 Model parameters

For most model parameters we used commonly cited values (Table 1). Since estimates of the degree of assortativeness in the population of MSM in the Netherlands are not available, we considered intermediate mixing ($\omega = 0.5$). The initial population size, N_0, was adjusted so that the steady-state population size in the absence of PrEP was about 190 000 individuals [2]. The average number of new partners per year, c_l, was determined from Rutgers World Population Foundation sexual behavior data from 2006 for MSM in the Netherlands [3] using the method described in detail in Ref. [1]. We used population stratification by risk from Ref. [1] where three groups with highest risk were grouped into one. The percentages of MSM in the four risk groups were 45.1%, 35.3%, 12.5% and 7.1%. The average number of new partners per year (range) was 0.13 (00.45), 1.43 (0.453.35), 5.44 (3.358.88) and 18.21 (8.88500), respectively.

We related annual ART uptake rate, τ, to the percentage of infected individuals starting ART within one year, τ^*, as $\tau^* = [1-\exp(-\tau \times 1year)]\,100\%$. Similarly, we related ART dropout rate, ϕ, to the percentage of treated individuals who drop out from ART within one year, ϕ^*, as $\phi^* = [1-\exp(-\phi \times 1year)]\,100\%$. Finally, PrEP uptake rate in risk group l, K_l^{on}, was related to the percentage of HIV-negative individuals in risk group l starting to use PrEP within one year, K_l^{on*}, as $K_l^{on*} = [1-\exp(-K_l^{on} \times 1year)]\,100\%$. All the results in the main text were presented in terms of annual ART, τ^*, and PrEP, K_l^{on*}, uptake percentages (range 0—100%).

The probability of transmission per partnership, λ, and annual ART uptake percentage τ^* were chosen so that in the absence of PrEP HIV prevalence was 8% [12] and ART coverage was 80% [11]. The baseline value for PrEP effectiveness $(1-\Omega)$ was 86% [13, 14]. The baseline duration of taking PrEP $(1/K_l^{off})$ was 5 years [2]. The sensitivity analyses for these two parameters were shown in the main text. The baseline value for infectivity of individuals who acquired HIV while taking PrEP was half the value of infectivity of individuals with primary infection who did take PrEP ($\epsilon^P = h_1/2$). We assumed that

annual ART uptake percentage for individuals who were infected on PrEP, τ_l^{P*}, was 95%. The sensitivity analyses for ϵ^P and τ_l^{P*} for the scenario of PrEP uptake in the highest risk group are shown in Figure 2.

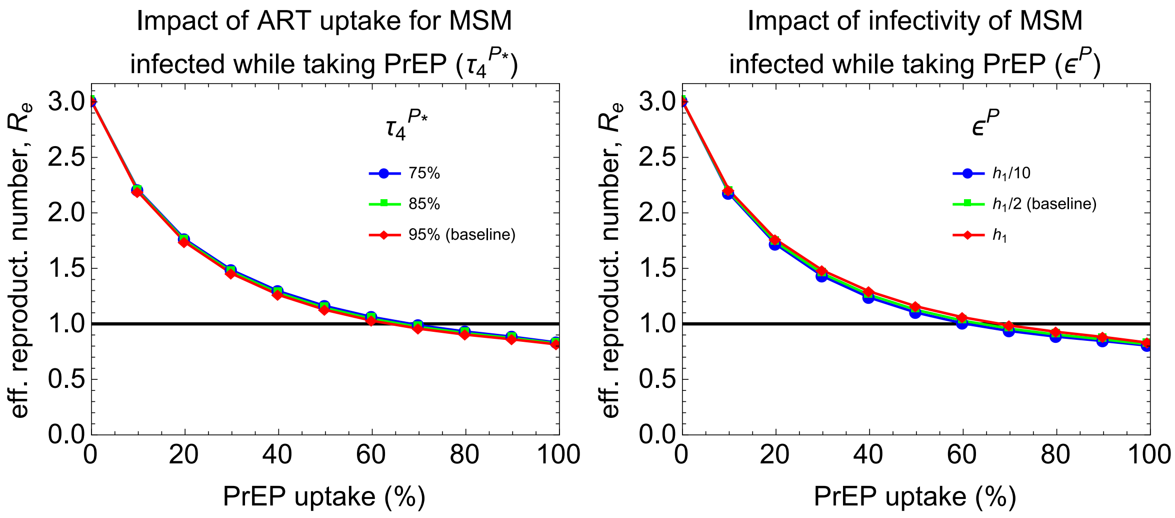

Figure 2: Sensitivity analyses for annual ART uptake percentage by individuals who were infected while taking PrEP (left) and infectivity of individuals who were infected while taking PrEP (right) for the scenario when PrEP is taken in the highest risk group only. The model results for the effective reproduction number were insensitive to variation of both parameters. The baseline parameter values indicated in the figure were used in the main text.

PrEP parameter specification for uptake in the highest risk group:

- $K_l^{on} = 0$, $K_l^{off} = 0$, $\tau_l^P = 0$ for $l = 1, 2, 3$.
- K_4^{on}, K_4^{off}, τ_4^P were varied as described in Table 1.

PrEP parameter specification for uptake in two groups with highest risk:

- $K_l^{on} = 0$, $K_l^{off} = 0$, $\tau_l^P = 0$ for $l = 1, 2$.
- $K_3^{on} = K_4^{on}$, $K_3^{off} = K_4^{off}$, $\tau_3^P = \tau_4^P$ were varied as described in Table 1.

4 Additional results

In Figure 3 we extended the results shown in Figure 1 C in the main text to include a wider range of PrEP uptake percentages. In this scenario, HIV elimination occurred when PrEP uptake exceeded 64% (see Figure 1 A in the main text). In Figure 3 we showed the

Table 1: Model parameters and their baseline values.

Notation	Baseline value, unit	Description
ρ_k	$\rho_1 = 1/0.142$ yr^{-1}<br>$\rho_2 = 1/8.439$ yr^{-1}<br>$\rho_3 = 1/1.184$ yr^{-1}	Rate of transition from stage k to stage $k+1$ for untreated individuals ($k = 1, 2, 3$) [4, 5, 6, 7, 8]
ρ_4	$\rho_4 = 1/1.316$ yr^{-1}	HIV related mortality for untreated individuals [4, 5, 6, 7, 8]
γ_k	$\gamma_1 = 1/8.21$ yr^{-1}<br>$\gamma_2 = 1/54.0$ yr^{-1}<br>$\gamma_3 = 1/2.463$ yr^{-1}	Rate of transition from stage k to stage $k+1$ for treated individuals ($k = 1, 2, 3$) [4, 6, 9]
γ_4	$\gamma_4 = 1/2.737$ yr^{-1}	HIV related mortality for treated individuals [4, 6, 9]
h_k	$h_1 = 0.62$<br>$h_2 = 0.12$<br>$h_3 = 0.642$<br>$h_4 = 0.0$	Infectivity of untreated individuals in stage k of infection ($k = 1, 2, 3, 4$) [4, 7, 8]
N_0	210000	Initial population size; adjusted so that the steady-state population size in the absence of PrEP was 190 000 [2]
ϵ	0.01	Infectivity of treated individuals [4, 10]
ω	[0,1]<br>0.5 (baseline)	Mixing parameter ($\omega = 0$ – assortative and $\omega = 1$ – proportionate mixing)
μ	$1/45$ yr^{-1}	Rate of recruitment to sexually active population [1]
q_l, $l = 1, \dots, 4$	$q_1 = 0.451$<br>$q_2 = 0.353$<br>$q_3 = 0.125$<br>$q_4 = 0.071$	Initial population fractions in risk group l [1]
c_l (range), $l = 1, \dots, 4$	$c_1 = 0.13$ (0—0.45) yr^{-1}<br>$c_2 = 1.43$ (0.45—3.35) yr^{-1}<br>$c_3 = 5.44$ (3.35—8.88) yr^{-1}<br>$c_4 = 18.21$ (8.88—500) yr^{-1}	Average number of new partners per year in risk group l (range) [1]

Notation	Baseline value, unit	Description
ϕ^*	5%	Annual ART droupout percentage [1]
ϕ	$-\ln[1-5\%/100\%]$ yr^{-1}	Annual ART dropout rate [1]
τ^*	$[0\%, 100\%)$, 30% (baseline)	Annual ART uptake percentage; chosen so that without PrEP ART coverage was 80% [11]
τ	$-\ln[1-\tau^*/100\%]$ yr^{-1}	Annual ART uptake rate
λ	0.25	Transmission probability per partnership; chosen so that without PrEP HIV prevalence was 8% [12]
$1-\Omega$	0.86 (baseline)<br>[0.75-0.95] (sensit. analysis)	PrEP effectiveness [13, 14]
ϵ^P	$h_1/2$ (baseline)<br>$[0, h_1]$ (sensit. analysis)	Infectivity of MSM infected on PrEP
$1/K_l^{off}$	5 years (baseline)<br>[1 yr,5 yrs] (sensit. analysis)	Average duration of taking PrEP in risk group l [2]
K_l^{on*}	$[0\%, 100\%)$	Annual PrEP uptake percentage in risk group l
K_l^{on}	$-\ln[1-K_l^{on*}/100\%]$ yr^{-1}	Annual PrEP uptake rate in risk group l
τ_l^{P*}	$[30\%, 100\%)$ (sensit. analysis)<br>95% (baseline)	Annual ART uptake percentage for MSM in risk group l infected on PrEP
τ_l^P	$-\ln[1-\tau_l^{P*}/100\%]$ yr^{-1}	Annual ART uptake rate for MSM in risk group l infected on PrEP

time-dependent dynamics of HIV prevalence for PrEP uptakes above this threshold, viz for uptakes of 74%, 84%, 94% and 99%. The results indicate that above the threshold the higher PrEP uptake is the faster HIV elimination is achieved. If PrEP uptake is below the threshold, the higher uptake leads to higher reduction in HIV prevalence within a given time frame.

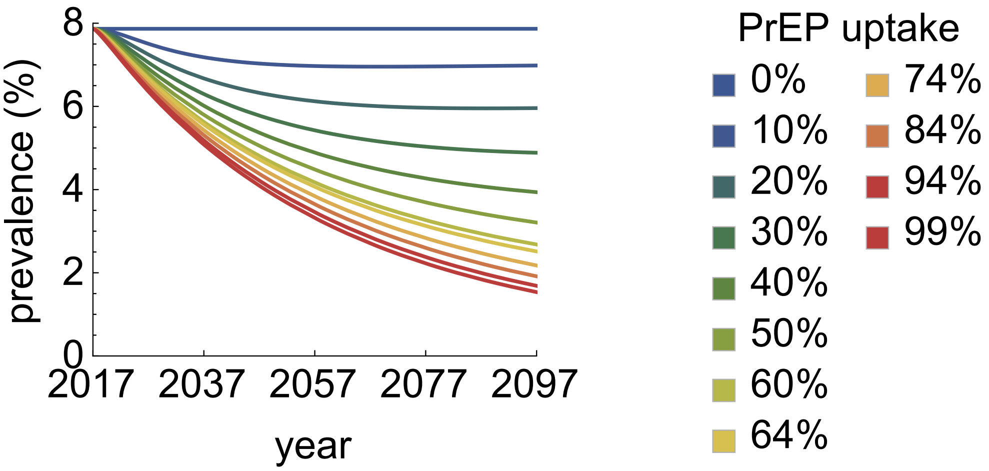

Figure 3: Impact of PrEP uptake in the highest risk group on the time-dependent dynamics of HIV prevalence for increasing levels of PrEP uptake and baseline ART. PrEP uptake was defined as the percentage of highest-risk MSM initiating PrEP within one year. The elimination occurred when PrEP uptake exceeded 64%. The results are extended from Figure 1 C in the main text to include PrEP uptakes above this threshold, viz for uptakes of 74%, 84%, 94% and 99%.

References

[1] G. Rozhnova, M. F. S. van der Loeff, J. C. M. Heijne, and M. E. Kretzschmar, “Impact of heterogeneity in sexual behavior on effectiveness in reducing HIV transmission with test-and-treat strategy,” *PLoS Comput. Biol.*, vol. 12, p. e1005012, 2016.

[2] B. E. Nichols, C. A. B. Boucher, M. van der Valk, B. J. A. Rijnders, and D. A. M. C. van de Vijver, “Cost-effectiveness analysis of pre-exposure prophylaxis for HIV-1 prevention in the Netherlands: a mathematical modelling study,” *Lancet Infectious Diseases*, vol. 16, pp. 1423–1429, 2016.

[3] R. N. Group, *Sexual health in the Netherlands*. Delft: Eburon, 2006.

[4] M. E. Kretzschmar, M. F. Schim van der Loeff, P. J. Birrell, D. De Angelis, and R. A. Coutinho, “Prospects of elimination of HIV with test-and-treat strategy,” *Proceedings of the National Academy of Sciences USA*, vol. 110, p. 1553815543, 2013.

[5] C. G. on AIDS Incubation, H. S. including the CASCADE EU Concerted Action. Concerted Action on SeroConversion to AIDS, and D. in Europe., “Time from HIV-1 seroconversion to AIDS and death before widespread use of highly-active antiretroviral therapy: a collaborative re-analysis,” *Lancet*, vol. 355, pp. 1131–1137, 2000.

[6] P. J. Birrell, A. M. Presanis, D. De Angelis, and T. C. Collaboration., “Multi-state models of HIV progression in homosexual men: an application to the CASCADE collaboration,” tech. rep., MRC Biostatistics Unit, 2012.

[7] T. D. Hollingsworth, R. M. Anderson, and C. Fraser, “HIV-1 transmission, by stage of infection,” *Journal of Infectious Diseases*, vol. 198, pp. 687–693, 2008.

[8] S. E. Bellan, J. Dushoff, A. P. Galvani, and L. A. Meyers, “Reassessment of HIV-1 acute phase infectivity: accounting for heterogeneity and study design with simulated cohorts,” *PLoS Medicine*, vol. 12, p. e1001801, 2015.

[9] T. C. C. C. A. on SeroConversion to AIDS and D. in Europe., “Survival after introduction of HAART in people with known duration of HIV-1 infection,” *Lancet*, vol. 355, pp. 1158–1159, 2000.

[10] R. M. Granich, C. F. Gilks, C. Dye, K. M. De Cock, and B. G. Williams, “Universal voluntary HIV testing with immediate antiretroviral therapy as a strategy for elimination of HIV transmission: a mathematical model,” *Lancet*, vol. 373, pp. 48–57, 2009.

[11] A. van Sighem, S. Boender, F. Wit, C. Smit, A. Matser, and P. Reiss, “HIV monitoring report 2017. Human Immunodeficiency Virus (HIV) infection in the Netherlands.” `https://www.hiv-monitoring.nl/index.php/download_file/view/1066/133/`, 2017.

[12] E. L. M. Op de Coul, I. Schreuder, S. Conti, A. van Sighem, M. Xiridou, M. G. Van Veen, and J. C. M. Heijne, “Changing patterns of undiagnosed HIV infection in the Netherlands: who benefits most from intensified HIV test and treat policies?,” *PLoS One*, vol. 10, p. e0133232, 2015.

[13] S. McCormack, D. T. Dunn, M. Desai, D. I. Dolling, M. Gafos, and R. e. a. Gilson, “Pre-exposure prophylaxis to prevent the acquisition of HIV-1 infection (PROUD):

effectiveness results from the pilot phase of a pragmatic open-label randomised trial," *Lancet*, vol. 387, pp. 53–60, 2016.

[14] J.-M. Molina, C. Capitant, B. Spire, G. Pialoux, L. Cotte, and I. e. a. Charreau, "On-demand preexposure prophylaxis in men at high risk for HIV-1 infection," *The New England Journal of Medicine*, vol. 373, pp. 2237–2246, 2015.
